# ASTER, ALI and Hyperion sensors data for lithological mapping and ore minerals exploration

**DOI:** 10.1186/2193-1801-3-130

**Published:** 2014-03-07

**Authors:** Amin Beiranvand Pour, Mazlan Hashim

**Affiliations:** Institute of Geospatial Science & Technology (INSTeG), Universiti Teknologi Malaysia, 81310 UTM Skudai, Johor Bahru Malaysia

**Keywords:** ASTER, ALI, Hyperion, Remote sensors, Ore minerals exploration, Lithological and structural mapping

## Abstract

This paper provides a review of the Advanced Spaceborne Thermal Emission and Reflection Radiometer (ASTER), Advanced Land Imager (ALI), and Hyperion data and applications of the data as a tool for ore minerals exploration, lithological and structural mapping. Spectral information extraction from ASTER, ALI, and Hyperion data has great ability to assist geologists in all disciplines to map the distribution and detect the rock units exposed at the earth’s surface. The near coincidence of Earth Observing System (EOS)/Terra and Earth Observing One (EO-1) platforms allows acquiring ASTER, ALI, and Hyperion imagery of the same ground areas, resulting accurate information for geological mapping applications especially in the reconnaissance stages of hydrothermal copper and gold exploration, chromite, magnetite, massive sulfide and uranium ore deposits, mineral components of soils and structural interpretation at both regional and district scales. Shortwave length infrared and thermal infrared bands of ASTER have sufficient spectral resolution to map fundamental absorptions of hydroxyl mineral groups and silica and carbonate minerals for regional mapping purposes. Ferric-iron bearing minerals can be discriminated using six unique wavelength bands of ALI spanning the visible and near infrared. Hyperion visible and near infrared bands (0.4 to 1.0 μm) and shortwave infrared bands (0.9 to 2.5 μm) allowed to produce image maps of iron oxide minerals, hydroxyl-bearing minerals, sulfates and carbonates in association with hydrothermal alteration assemblages, respectively. The techniques and achievements reviewed in the present paper can further introduce the efficacy of ASTER, ALI, and Hyperion data for future mineral and lithological mapping and exploration of the porphyry copper, epithermal gold, chromite, magnetite, massive sulfide and uranium ore deposits especially in arid and semi-arid territory.

## Introduction

Remote sensing technology has been used in diverse aspects of Earth sciences, geography, archeology and environmental sciences. Earth scientists have focused on global experiences in environmental geology, mineral and hydrocarbon exploration using remote sensing data (Kucukkaya 2004; Hellman and Ramsey 2004; Galvao et al., 2005; Watts and Harris 2005; Vaughan et al. 2005; Aminzadeh and Samani, 2006; Lammoglia and Filho 2011; Shi et al., 2012; Petrovic et al. 2012; van Ruitenbeek et al. 2012).

Recognizing hydrothermally altered minerals and lithological mapping through remote sensing instruments have been widely and successfully used for the exploration of epithermal gold, porphyry copper, massive sulfide, chromite, magnetite and uranium ore deposits (Velosky et al. 2003; di Tommaso and Rubinstein 2007; Rajesh 2008; Van Ruitenbeek et al. 2012; Zhang et al. 2007; Goetz 2009; Azizi et al., 2010; Ramadan and Abdel Fattah 2010; Pour et al. 2011; Pour and Hashim 2011a, Pour and Hashim 2012b, Pour and Hashim 2011c, Pour and Hashim 2011d; Bedini 2011; Amer et al., 2012; Rajendran et al. 2011, Rajendran et al. 2012). This review emphasizes on geological applications of the Advanced Spaceborne Thermal Emission and Reflection Radiometer (ASTER), Advanced Land Imager (ALI), and Hyperion remote sensors in the field of ore minerals exploration, lithological and structural mapping. Digital image processing techniques aid to extract required spectral information for geological applications are also elaborated in this paper.

In the initial stage of remote sensing technology development (1970s), geological mapping and mineral exploration were among the most prominent applications (Rowan et al. 1974; Rowan et al., 1977; Goetz et al., 1983; Abrams et al. 1983). Multispectral and hyperspectral remote sensing sensors were used for geological applications, ranging from a few spectral bands to more than 100 contiguous bands, covering the visible to the shortwave infrared regions of the electromagnetic spectrum (Abrams et al. 1983; Rowan and Wetlaufer 1981; Crowley et al., 1989; Spatz and Wilson, 1995; Clark et al. 1991; Crosta et al. 1998, Kruse et al., 1999; Goetz, 2009; van der Meer et al., 2012).

Landsat Multi-Spectral Scanner (MSS), Landsat Thematic Mapper (TM) and Syste`m Pour l’Observation de la Terre (SPOT) with four to seven spectral bands have been used for regional scales of geological mapping (Goetz et al., 1983; Sultan et al., 1987; Tangestani and Moore, 2000; Kavak 2005; Kargi 2007). HyMap and the Airborne Visible/IR Image Spectrometer (AVIRIS) hyperspectral sensors with 126 to 224 contiguous bands were used to provide information about hydrothermal alteration minerals on the Earth’s surface (Clark et al., 1991; Cocks et al., 1998; Kruse et al. 1999; Abdelsalam and Stern, 2000; Perry, 2004; Hellman and Ramsey 2004). Several investigations have discovered that remote sensing hyperspectral sensors are capable to map spectrally distinct hydrothermal alteration minerals (Crowley et al., 1989; Crowley and Clark, 1992; Kruse et al. 1993; Boardman et al., 1995; Crosta et al., 1998; Cocks et al., 1998; Kruse et al. 1999; Kruse et al., 2003; Gersman et al., 2008; Bedini et al., 2009).

Landsat Thematic Mapper /Enhanced Thematic Mapper^+^ (TM/ETM^+^) image has been used for detecting alteration mineral assemblages associated with epithermal gold and porphyry copper mineralization and lithological mapping applications. Shortwave infrared bands (bands 5 and 7) of TM/ETM^+^ have been used as a tool to identify hydroxyl-bearing minerals in the reconnaissance stages of copper/gold exploration (Rowan et al., 1977; Podwysocki et al., 1984; Crowley et al. 1989; Okada et al., 1993; Sabins, 1996; Sabins, 1997; Abdelsalam and Stern, 2000). Band ratio of 5/7 is sensitive to hydroxyl (OH) minerals, which are found in the alteration zones (Kusky and Ramadan, 2002; Inzana et al. 2003; Aydal et al., 2007; Rajesh, 2008; Ramadan and Abdel Fattah, 2010).

Hyperspectral sensors such as HyMap and the Airborne Visible/IR Image Spectrometer (AVIRIS) with more than 100 continuous bands in shortwave infrared region have been also used to obtain accurate information about hydrothermal alteration mineral assemblages (Cocks et al., 1998; Kruse et al., 1999; Kruse and Boardman, 2000; Gersman et al., 2008; Bedini et al., 2009; Bedini 2009; Goetz, 2009; Bedini 2011). Expensive mobilization and small coverage and not readily available data are problems associated with airborne-based hyperspectral data for geological mapping applications (Smailbegovic and Taranik, 1999).

The Advanced Spaceborne Thermal Emission and Reflection Radiometer (ASTER) remote sensor has sufficient spectral resolution in the shortwave length infrared radiation bands for mapping hydrothermal alteration mineral zones associated with porphyry copper and epithermal gold mineralization (Pour and Hashim, 2012a). Since 2000, ASTER data have been widely and successfully used in lithological mapping and mineral exploration (Pour et al., 2011; Pour and Hashim, 2011a, 2011b, 2011c, 2011d, 2012a, 2012b; Haselwimmer et al., 2011; Mars and Rowan, 2011; Bedini 2011; Vicente and Filho 2011; Tangestani et al., 2011; Rajendran et al., 2011, 2012; Amer et al., 2012; Zoheir and Emam 2012).

ALI has six unique wavelength channels spanning the visible and near infrared (0.4-1.0 micrometer (μm)). Because of their respective band center positions, ALI is especially useful for discriminating among ferric-iron bearing minerals in the standpoint of geologic mapping applications (Hubbard et al., 2003; Hubbard and Crowley 2005).

Hyperion shortwave infrared bands (2.0 to 2.5 μm) can uniquely identify and map hydroxyl-bearing minerals, sulfates and carbonates in the hydrothermal alteration assemblages (Kruse et al., 2003; Gersman et al., 2008; Bishop et al., 2011). First subset of visible and near infrared bands between 0.4 and 1.3 μm can also be used to highlight iron oxide minerals (Bishop et al., 2011).

The near coincidence of EO1 and EOS/Terra platforms allows obtaining images of the same ground areas, resulting comprehensive remote sensing information for the reconnaissance stages of mineral exploration. A comparison approach is also to be used between ASTER, ALI and Hyperion imagery in the field of mineral exploration. Spectral information extraction from ASTER, ALI and Hyperion data has a great ability to assist economic geologists for exploring high economic-potential copper and gold mineralization zones, massive sulfide, chromite, magnetite and uranium ore deposits especially in the arid and semi-arid realms of the Earth.

## Visible near-infrared, shortwave infrared and thermal infrared spectra of hydrothermal alteration minerals

The ability to discriminate between hydrothermally altered and unaltered rocks are considerable in mineral exploration studies. In the region of solar reflected light (0.325 to 2.5 μm), many minerals demonstrate diagnostic absorption features due to vibrational overtones, electronic transition, charge transfer and conduction processes (Hunt 1977; Hunt and Ashley 1979; Clark et al., 1990; Cloutis 1996). Hydrothermally altered rocks are frequently indicated by iron oxide, clay, carbonate, and sulfate minerals, which produce diagnostic absorption signatures throughout the visible and near infrared (VNIR) and shortwave infrared (SWIR) regions.

Iron oxide/hydroxide minerals such as limonite, jarosite and hematite tend to have spectral absorption features in the visible to middle infrared from 0.4 to 1.1 μm of the electromagnetic spectrum (Hunt and Salisbury 1974; Hunt 1977; Hunt and Ashley 1979). Iron oxides are one of the important mineral groups that are associated with hydrothermally altered rocks over porphyry copper bodies (Sabins 1999). Electronic processes produce absorption features in the visible and near infrared radiation (0.4 to 1.1 μm) due to the presence of transition elements such as Fe^2+^, Fe^3+^ and often substituted by Mn, Cr, and Ni in the crystal structure of the minerals (Hunt 1977; Hunt and Ashley 1979). Iron oxide/hydroxide minerals produce during supergene alteration and render characteristic yellowish or reddish color to the altered rocks, which are collectively termed gossan (Abdelsalam and Stern 2000; Xu et al., 2004).

The shortwave infrared radiation is the best spectral region of the electromagnetic spectrum for sensing various aspects of hydrothermal alteration zones. Hydroxyl-bearing minerals including clay and sulfate groups as well as carbonate minerals present diagnostic spectral absorption features due to vibrational processes of fundamental absorptions of Al–O–H, Mg–O–H, Si–O–H, and CO_3_ groups in the shortwave infrared radiation region, and thus this wavelength region is the best to explore and map hydrothermal alteration zones. The most important characteristics of the SWIR wavelength region are to identify and map the spatial distribution of hydrothermal alteration minerals containing OH groups (Huntington 1996).

Phyllosilicates, including Al-Si-(OH) and Mg-Si-(OH) bearing minerals such as kaolinite, montmorillonite, muscovite, illite, talc and chlorite, and sorosilicate group, including Ca-Al-Si-(OH) bearing minerals such as epidote group, and OH-bearing sulfates, including alunite and gypsum, and also carbonates can be identified by virtue of their spectral characteristics in shortwave infrared radiation region (Hunt 1977; Hunt and Ashley,^1979^; James et al., 1988; Clark et al., 1990).

Therefore, the remote sensing shortwave infrared radiation data are capable in identifying of hydrothermal alteration mineral assemblages including: (i) mineralogy generated by the passage of low PH fluids (alunite and pyrophylite); (ii) Al-Si-(OH) and Mg-Si-(OH)-bearing minerals, including kaolinite and mica and chlorite groups; and (iii) Ca-Al-Si-(OH) bearing minerals such as epidote group, as well as carbonate group (calcite and dolomite).

**Figure 1:**
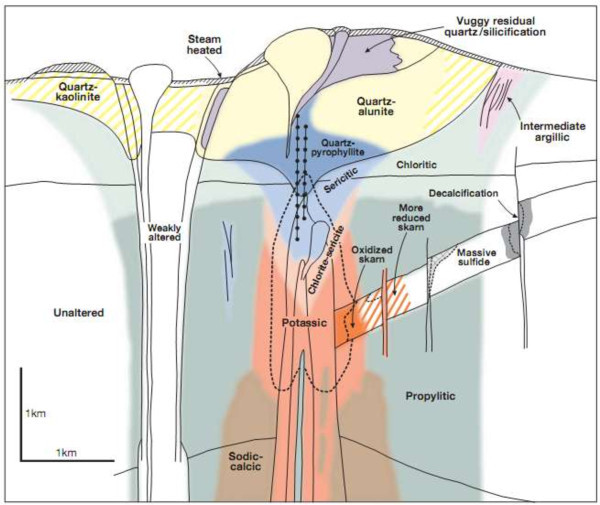
**Generalized alteration-mineralization zoning pattern for porphyry copper deposit (Sillitoe** 2010 **).**

In the idealized porphyry copper deposit model (Figure 1), a core of quartz and potassium-bearing minerals, mostly potassium feldspar and biotite, is surrounded by multiple hydrous zones of alteration minerals (Lowell and Guilbert 1970; Sillitoe 2010). The hydrous zones are characterized by mineral assemblages, which contain at least one mineral that exhibits diagnostic spectral absorption features in the visible near-infrared (VNIR) through the short-wave infrared (SWIR; 0.4–2.5 μm) and (or) the thermal-infrared (TIR; 8.0–14.0 μm) wavelength regions (Abrams and Brown 1984; Hunt and Ashley 1979; Spatz and Wilson 1995).

**Figure 2:**
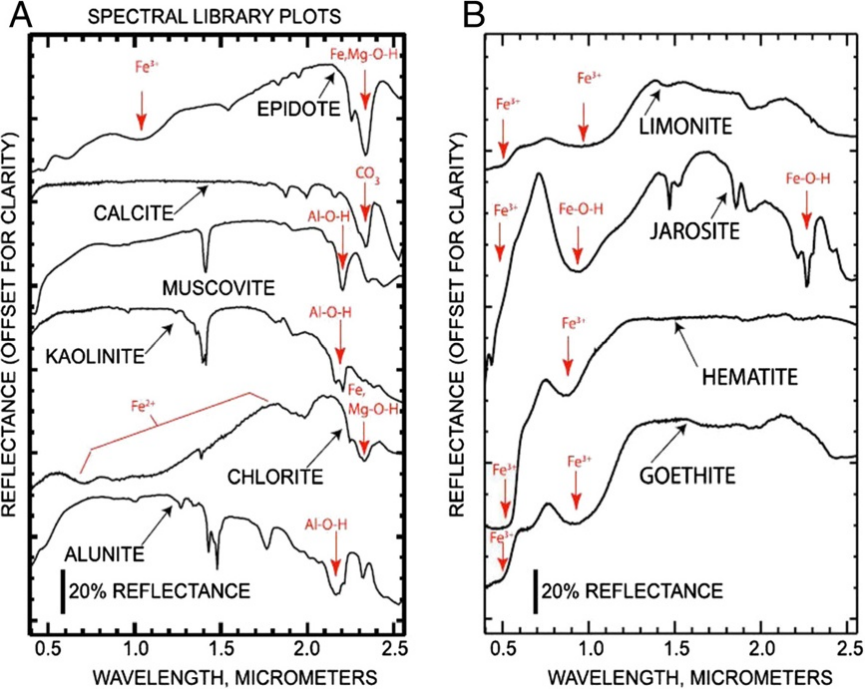
**Laboratory spectra of hydrothermal alteration minerals. (A)** Laboratory spectra of clay minerals. **(B)** Laboratory spectra of iron oxide minerals (Clark et al., 1993).

Sericitically-altered rocks typically contain sericite, a fine-grained form of muscovite that has a distinct Al-OH absorption feature at 2.2 μm and a less intense absorption feature at 2.35 μm (Figure 1A; Abrams and Brown 1984; Spatz and Wilson 1995). Kaolinite and alunite are typical constituents of advanced argillic alteration that exhibit Al-OH 2.165 μm and 2.2 μm absorption features (Figure 2A; Hunt 1977; Hunt and Ashley 1979; Rowan et al., 2003). Although less common than alunite or kaolinite, advanced argillic-altered rocks can also contain pyrophyllite which has an intense 2.165 μm Al-O-H absorption feature. Propylitically-altered rocks typically contain varying amounts of chlorite, epidote and calcite, which exhibit Fe, Mg-O-H and CO_3_ 2.31–2.33 μm absorption features (Figure 2A; Rowan and Mars 2003).

VNIR–SWIR spectra of epidote and chlorite also exhibit broad, prominent Fe^2+^ absorption spanning the 0.62 to 1.65 μm region (Figure 2A). Supergene altered deposits typically contain alunite, kaolinite, limonite, goethite, hematite and jarosite (Gustafson and Hunt 1975; Di Tommaso and Rubinstein 2007). Goethite, hematite and limonite have strong Fe^3+^ absorption features at 0.97–0.83 μm and 0.48 μm (Figure 2B; Hunt et al., 1971a). Jarosite has Fe-O-H absorption features at 0.94 μm and 2.27 μm (Figure 2B; Hunt et al., 1971b).

Hydrothermal silicification accompanies mineralization in many metal deposits, thus, the identification and mapping of quartz in rocks composed mainly of other minerals is of great value for exploration and assessments of resource potential (Rockwell and Hofstra 2008). Hydrothermally altered silica-rich rocks associated with porphyry copper deposits consist primarily of quartz veins, silica lithocaps, or silicified deposits (Titley 1972). Hydrothermal silica minerals typically consist of quartz, opal and chalcedony. TIR emissivity spectra illustrate that quartz and opal contain a prominent restrahlen feature in the 9.1 μm region. Therefore, sericitic alteration zone, advanced argillic alteration zone, propylitic alteration assemblages, hydrothermal silica-rich rocks and supergene altered deposits can be mapped using VNIR, SWIR and TIR spectral features.

Quartz and carbonate minerals are spectrally characterized by strong vibrational absorption features within the 8–14 μm (Salisbury and D’Aria 1992a; Hook et al., 1999). The emissivity absorption features of quartz at 8.3 and 9.1 μm are related to fundamental asymmetric Si-O stretching vibrations (reststrahlen bands). The reststrahlen bands of quartz are the strongest of any silicate mineral (Salisbury and D’Aria 1992b). The emissivity absorption features of calcite and dolomite at 11.3 μm are related to out-of-plane bending modes of the CO3 ion (Clark 1999). Note that dolomite exhibits a greater decrease in emissivity between 10.7 and 11.3 μm than calcite. This characteristic is caused by the greater width and shorter wave-length position of the bending feature of dolomite at 11.15 μm relative to the bending feature of calcite at 11.27 μm (Rockwell and Hofstra 2008).

Previous studies have demonstrated the identification of specific hydrothermal alteration minerals, such as alunite, kaolinite, calcite, dolomite, chlorite, quartz, talc and muscovite, as well as mineral groups, through the analysis of remote sensors depending on their spatial and spectral resolutions (Hewson et al., 2001; Rowan and Mars 2003; Rowan et al., 2003; Kruse et al., 2003; Junek 2004; Hellman and Ramsey 2004; Galvao et al., 2005; Mars and Rowan 2006; Rowan et al., 2006; Ducart et al., 2006; Di Tommaso and Rubinstein 2007; Gersman et al., 2008; Sanjeevi 2008; Bedini et al., 2009; Azizi et al., 2010; Gabr et al., 2010; Mars and Rowan 2010; Kratt et al., 2010; Pour et al., 2011; Pour and Hashim 2011a, 2011b, 2011c, 2011d; Oztan and Suzen 2011; Haselwimmer et al., 2011; Mars and Rowan 2011; Bedini 2011; Vicente and Filho 2011; Amer et al., 2012).

## Remote sensors

Recently, the launch of sophisticated remote sensors developed by National Aeronautics and Space Administration (NASA) on the earth orbiter spacecraft such as the Earth Observing System (EOS)/Terra and the Earth Observing-1 (EO-1) platforms, has created opportunities for improving the quality and reducing the cost of remote sensing data. The EOS/Terra platform was launched into a near-polar orbit at an altitude of 702 km on 18 December 1999. The EOS/Terra is an advanced spaceborne platform carrying three sophisticated sensor consisting of (i) the Moderate Resolution Imaging Spectrometer (MODIS); (ii) the Multiangle Imaging SpectroRadiometer (MISR); and (iii) the Advanced Spaceborne Thermal Emission and Reflection Radiometer (ASTER) (Pieri and Abrams 2004).

**Figure 3:**
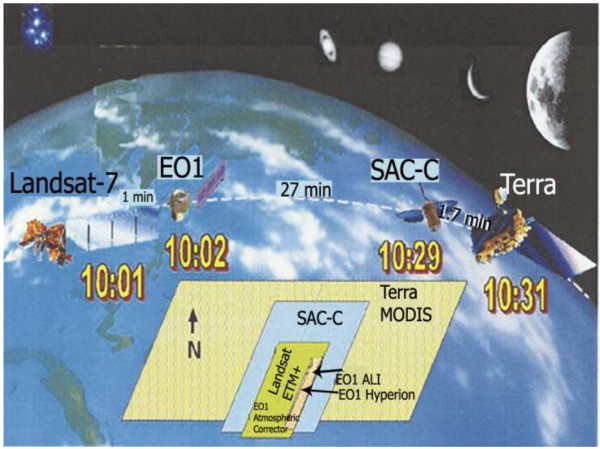
**A view of the “morning constellation” consists of Landsat-7, EO1, SAC-C and Terra platforms (Ungar et al.,** 2003 **).**

The Earth Observing-1 (EO-1) satellite was launched on 21 November of 2000 as part of NASA’s New Millennuim Program (NMP) technology path-finding activities to enable more effective (and less costly) hardware and strategies for meeting earth science mission needs in the 21st century. The EO-1 platform includes three of the most advanced remote sensing instruments (i) The Advanced Land Imager (ALI); (ii) Hyperion; and (iii) The Linear Etalon Imaging Spectral Array (LEISA) Atmospheric Corrector (LAC). These sensors can be used in a variety of scientific disciplines (Beck 2003; Ungar et al., 2003). The EO-1 platform orbits in a ground track coverage that is one minute later than Landsat-7 Thematic Mapper. Following EO-1, in nearly the same orbit, are Satelite de Aplicanciones Cientificas (SAC-C; an Argentinean satellite) and EOS/Terra. Landsat-7 platform passes over the equator in descending node at 10:01 AM (Figure 3) (Folkman et al., 2001; Ungar et al., 2003).

### Aster

**Figure 4:**
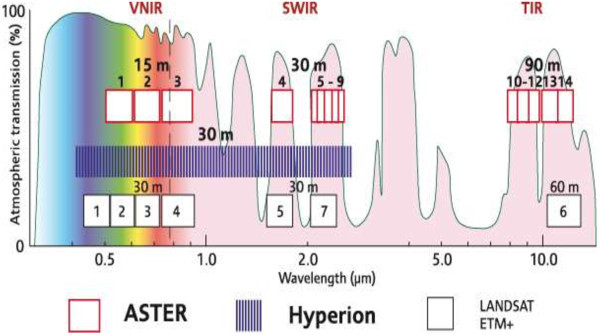
**Hyperion and ASTER spectral bands compared to Landsat-7 ETM** ^**+**^ **(Waldhoff et al.,** 2008 **).**

The Advanced Spaceborne Thermal Emission and Reflection Radiometer (ASTER) is a high spatial, spectral and radiometric resolution multispectral remote sensing sensor. It was launched on NASA’s Earth Observing System AM-1 (EOS AM-1) polar orbiting spacecraft in December 1999. EOS AM-1 spacecraft operates in a near polar, sun-synchronous circular orbit at 705 km altitude. The recurrent cycle is 16 days, with additional 4 day repeat coverge due to its off-nadir pointing capabilities. ASTER is a cooperative effort between the Japanese Ministry of Economic Trade and Industry (METI) and National Aeronautics and Space Administration (NASA). It consists of three separate instrument subsystems, which provide observation in three different spectral regions of the electromagnetic spectrum, including visible and near infrared (VNIR), shortwave infrared (SWIR) and thermal infrared (TIR) (Figures 3 and 4) (Abrams et al., 2004; Pour and Hashim 2012a).

The VNIR subsystem has three recording channels between 0.52 and 0.86 μm and an additional backward-looking band for stereo construct of Digital Elevation Models (DEMs) with a spatial resolution of up to 15 m. The SWIR subsystem has six recording channels from 1.6 to 2.43 μm, at a spatial resolution of 30 m, while the TIR subsystem has five recording channels, covering the 8.125 to 11.65 μm wavelength region with spatial resolution of 90 m. ASTER swath width is 60km (each individual scene is cut to a 60x60 km^2^ area) which makes it useful for regional mapping, though its off-nadir pointing capability extends its total possible field of view to up to 232 km. ASTER can acquire approximately 600 scenes daily, but is generally targeted and tasked without continuous operation unlike other multispectral sensors such as Landsat (Fujisada 1995; Abrams and Hook 1995; Yamaguchi et al., 1999; Abrams 2000; Yamaguchi et al., 2001; Abrams et al., 2004; Pour and Hashim 2012a). The performance characteristics of ASTER data are shown in Table 1. ASTER standard data products are discussed in detail by Pour and Hashim (Pour and Hashim 2012a).

**Table 1:** **The technical characteristics of ASTER data (Fujisada,** 1995 **; Yamaguchi et al.,** 1999 **)**

Subsystem	Band number	Spectral range (μm)	Radiometric resolution	Absolute accuracy (σ)	Spatial resolution
VNIR	1	0.52-0.60	NE Δρ ≤ 0.5%	≤ 4%	15 m
	2	0.63-0.69			
	3N	0.78-0.86			
	3B	0.78-0.86			
SWIR	4	1.600-1.700	NE Δρ ≤ 0.5%		
	5	2.145-2.185	NE Δρ ≤ 1.3%		
	6	2.185-2.225	NE Δρ ≤ 1.3%	≤ 4%	30 m
	7	2.235-2.285	NE Δρ ≤ 1.3%		
	8	2.295-2.365	NE Δρ ≤ 1.0%		
	9	2.360-2.430	NE Δρ ≤ 1.3%		
TIR	10	8.125-8.475		≤ 3K(200-240K)	
	11	8.475-8.825		≤ 2K(240-270K)	90 m
	12	8.925-9.275	NE ΔT ≤ 0.3 k	≤ 1K(270-340K)	
	13	10.25-10.95		≤ 2K(340-370K)	
	14	10.95-11.65			
Signal quantization levels					
Stereo base-to-height ratio		0.6 (along-track)			
Swath width		60 km			
Total coverage in cross-track direction by pointing			232 km		
Coverage interval		16 days			
Altitude		705 km			
MTF at Nyquist frequency		0.25 (cross-track)			
		0.20 (along-track)			
Band to band registration		Intra-telescope: 0.2 pixels			
Peak power		726 w			
Mass		406 kg			
Peak data rate		89.2 Mbps			

Band number 3N refers to the nadir pointing view, whereas 3B designates the backward pointing view.

### ALI and Hyperion

The Advanced Land Imager (ALI) is a prototype for a new generation of Landsat-7 Thematic Mapper. The ALI provides multispectral data similar to that of the Enhanced Thematic Mapper Plus (ETM+) sensor on Landsat-7. The sensor maintains similar characteristics to Landsat-7 with a spatial resolution of 30 m; however, the swath width is 37 km as opposed to 185 km (Hearn et al., 2001; National Aeronautics and Space Administration, 2002, 2004; Wulder et al., 2008). ALI is a pushbroom sensor and has some additional bands in comparison with whiskbroom design of ETM^+^ sensor (Thome et al., 2003). The performance characteristics of the ALI and ETM^+^ are shown in Table 2. Additional bands in the ALI improved Signal-to-Noise Ratio (SNR) that is one of the most significant performance aspects of the ALI to increase the quality of data (Lencioni et al., 1999; Mendenhall et al. 2000; Thome et al., 2003; Lobell and Asner 2003).

**Table 2:** **The performance characteristics of the ALI and ETM** ^**+**^ **sensors (Bryant et al.,** 2003 **; Beck,** 2003 **; Lobell and Asner,** 2003 **)**

Sensors	Subsystem	Band number	Spectral range (μm)	Ground Resolution (m)	Swath Width (km)
ALI	VNIR	Pan	0.480-0.690	10	37
		1	0.433-0.453	30	
		2	0.450-0.515		
		3	0.525-0.605		
		4	0.633-0.690		
		5	0.775-0.805		
		6	0.845-0.890		
	SWIR	7	1.200-1.300		
		8	1.550-1.750		
		9	2.080-2.350		
ETM +	VNIR	Pan	0.520-0.900	14.25	185
		1	0.450-0.515	28.50	
		2	0.525-0.605		
		3	0.633-0.690		
		4	0.780-0.900		
	SWIR	5	1.550-1.750		
		7	2.090-2.350		
	TIR	6	10.45-12.50		

**Figure 5:**
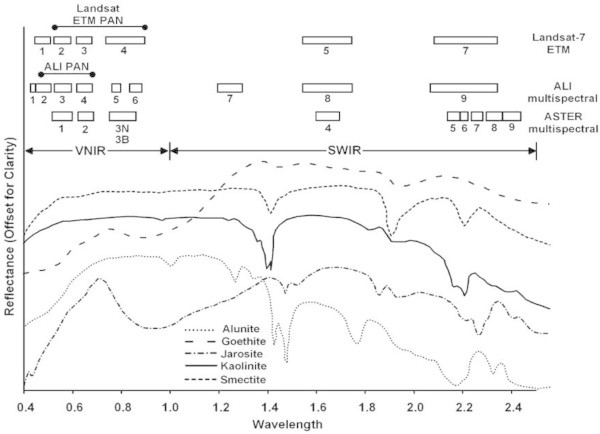
**The comparison of ALI, ETM** ^**+**^ **and ASTER spectral bandpasses on the subject of hydrothermal alteration mineral mapping (Hubbard and Crowley,** 2005 **)** **.**

ALI has 10 channels spanning the visible and near infrared (VNIR) to shortwave infrared (SWIR) (0.4-2.35 μm), one panchromatic, six VNIR and three SWIR. ALI VNIR data can be especially useful for detecting iron oxide minerals in the standpoint of geologic mapping applications (Hubbard et al., 2003; Hubbard and Crowley 2005). Figure 5 shows the comparison of ALI, ETM^+^ and ASTER spectral bandpasses on the subject of hydrothermal alteration mineral mapping (Hubbard and Crowley 2005).

Hyperion is the first advanced satellite hyperspectral sensor in commission across the spectral coverage from 0.4 to 2.5 μm and 10 nm spectral resolution. It is a pushbroom instrument with 242 spectral channels over a 7.6 km swath width, and 30m spatial resolution (Liao et al., 2000; Barry and Pearlman, 2001).

The system has two spectrometers and a single telescope. Spectrometers operate at visible and near infrared wavelength (approximately 0.4 to 1.0 μm) and at shortwave infrared wavelength (approximately 0.9 to 2.5 μm), respectively. The 242 total bands include the first 70 bands in the visible and near infrared region and the second 172 bands in the shortwave infrared region, 21 bands are located in a region of bands’ overlap between 0.9 and 1.0 micrometer (Folkman et al., 2001; Pearlman et al., 2003; Ungar et al. 2003; Beck 2003; Green et al., 2003, Goodenough et al., 2003).

Figure 4 depicts the Hyperion and ASTER spectral bands compared to Landsat-7 ETM^+^ (Waldhoff et al., 2008). The performance characteristics of Hyperion are shown in Table 3. The scientists’ range of interests that can achieve good benefit from Hyperion data include: (1) geology; (2) agriculture monitoring; (3) volcanic temperature measurement; (4) study of reef and coral bay health; (5) glaciological applications (Barry et al., 2002). Hyperion data have a signal-to-noise ratio about 161/1 in the visible and near infrared and 40/1 in the shortwave infrared region that somewhat limited the scientific applications of Hyperion data (Folkman et al., 2001; Thome et al., 2003).

**Table 3:** **The performance characteristics of the Hyperion sensor (Folkman et al.,** 2001 **)**

Sensor	Subsystem	Band number	Spectral range (μm)	Ground resolution (m)	Swath Width (km)	Radiometric precision (S/N)
Hyperion	VNIR	Continuous	0.400-1.000	30	7.6	161/1
	SWIR	Continuous	0.900-2.500	30	7.6	40/1

## Mapping lithology and ore minerals exploration using ASTER data

ASTER data have been extensively used for a wide range of geological applications, including lithological and structural mapping, ore minerals exploration, hydrocarbon prospecting and environmental geology. The use of ASTER data in lithological/structural mapping and ore minerals exploration particularly for porphyry copper, epithermal gold, chromite, magnetite, massive sulfide and uranium has increased in recent years. Accordingly, we review the applications of the ASTER data for lithological/structural mapping and ore minerals exploration purposes in detail here.

The capability of the ASTER multispectral data for geologic and alteration mineral mapping has been simulated for Mountin Fitton, South Australia (Hewson et al. 2001). This test site has been previously surveyed by visible-shortwave hyperspectral AMS (HyMap), Thermal Infrared Multispectral Scanner (TIMS) data and several field campaigns collecting relevant spectral measurements. They applied decorrelation stretch on simulated ASTER bands 3-2-1 to delineate drainage and vegetation, and band 13-12-10 for the identification of quartz rich areas. They also implemented Mixture Tuned Matched Filtering (MTMF - Boardman et al., 1995; Boardman 1998) method on the simulated ASTER SWIR bands to obtain spectrally unmixed end-members related to the rich areas of hydrothermally alteration mineral assemblages. Their results showed good accuracy with field spectral measurements, and compared well with HyMap and TIMS outputs that were collected previously for the study area.

Relative absorption-band depth (RBD - Crowley et al., 1989), Matched Filtering (MF - Harsanyi et al. 1994; Boardman et al., 1995) and Spectral Angle Mapper (SAM - Kruse et al., 1993) methods have been applied on ASTER data for differentiating calcitic, granodioritic, gneissic, granitic and quartzos rock units in Mountain Pass, California, USA. The results showed good similarity between the patterns of the identified rock units with geologic map of the study area (Rowan and Mars 2003).

ASTER band ratios and relative absorption band depth (RBD), Matched Filtering (MF) and Spectral Angle Mapper (SAM) methods have been also used for lithological mapping of the ultramafic complex in the Mordor Pound, NT, Australia (Rowan et al., 2005). Felsic and mafic-ultramafic rocks, alluvial-colluvial deposits and quartzose to intermediate composition rocks were discriminated and classified based on spectral absorption features of Al-OH and ferric-iron mineralogical groups for felsic rock, ferrous-iron and Fe, Mg-OH mineralogical absorption features for mafic-ultramafic rock using ASTER VNIR + SWIR bands. Additional Si-O spectral features were used to map more lithologic diversity within ultramafic complex and adjacent rocks such as mafic-gneisses, felsic-gneisses, intermediate composition rocks such as syenite and quartzite using ASTER TIR bands. ASTER SWIR and TIR data were used to produce regional-scale maps of Al-OH and Mg-OH/carbonate minerals and quartz content for the Broken Hill-Curnamona province of Australia (Hewson et al., 2005).

Chemical composition of quartzose, carbonate and silicate rocks have been detected using Quartz Index (QI), Carbonate Index (CI) and Mafic Index (MI) to ASTER-TIR in Mountain Yushishan, China and Mountain Fitton, Australia. These lithologic indices discriminated quartz, carbonate and mafic-ultramafic rocks, which were compatible well with published geologic map and field observation. They suggested that these lithologic indices can be one unified approach for lithological mapping of the Earth, especially in arid and semi-arid regions.

Principal Component Analysis (PCA - Singh and Harrison 1985) and supervised classification have been applied on visible near infrared and shortwave infrared ASTER bands to identify lithological units in the Western margin of the Kalahari Desert in Namibia. The processing of ASTER data demonstrated validation of the lithological boundaries defined on previous geological map, and also provided the information for characterizing new lithological units, which were previously unrecognized (Gomez et al., 2005).

Spectral Angle Mapper (SAM), Spectral Feature Fitting (SFF - Clark and Roush 1984) and Linear Spectral Umixing (LSU - Boardman 1989, 1992) methods have been employed on 14 ASTER bands for lithological mapping in the Neoproterozoic Allaqi-Heiani Suture, Southern Egypt (Qiu et al. 2006). Gabrro and mafic volcanic rocks, talk carbonate schist, granite and felsic meta-volcanic rocks, sand and wadi fill were detected using ASTER data.

Principal Component Analysis (PCA), Fast Fourier Transform (FFT - Gonzalez and Woods 2002) and Redundant Wavelet Transform (RWT - Brown 2000) techniques have been implemented on VNIR and SWIR bands of ASTER to identify ophiolite components consisting of talk carbonate schist, gabbro and serpentinite, as well as Neoproterozoic ductile structures to trace along-strike continuation in the Allaqi-Heiani Suture, southern Egypt (Ren and Abdelsalam 2006).

New ASTER band ratio images 4/7, 4/6 and 4/10 have been generated for lithological mapping in the Arabian–Nubian shield, the Neoproterozoic Wadi Kid area, Sinai, Egypt (Gad and Kusky 2007). These ASTER band ratios mapped the main rock units consisting of gneiss and migmatite, amphibolite, volcanogenic sediments with banded iron formation, meta-pelites, talc schist, meta-psammites, meta-acidic volcanics, meta-pyroclastics volcaniclastics, albitites and granitic rocks.

Principal Component Analysis (PCA), Minimum Noise Fraction (MNF - Green et al., 1988) techniques have been applied to VNIR + SWIR ASTER data for lithological mapping in Muslim Bagh ophiolite complex, Pakistan. The PCA discriminated metamorphic sole, sheeted dike complex, basalt and cherts, diabase dikes and gabbro bodies. The MNF transformed data detected sedimentary units, metamorphic sole, laterite, depleted harzburgite and diabase dikes/sills (Khan et al., 2007).

ASTER multispectral data have been integrated with the Airborne Visible/Infrared Imaging Spectrometer (AVIRIS) and EO-1 Hyperion hyperspectral data to extending hyperspectral signatures to regional scales mineral mapping and environmental monitoring in northern death valley, south-central California/Nevada, USA (Kruse and Perry 2007). The AIG-Developed hyperspectral analysis approach (Kruse and Boardman 2000) was applied to ASTER data. Their results indicated that the AIG methods are not only a way to analyze hyperspectral data, while can achieved accurate resultants when selectively employed on ASTER multispectral data. Moreover, AIG methods can also provide a consistent way to extract spectral information from hyperspectral and multispectral data without a priori knowledge or requiring ground observations.

ASTER data have been processed for granitoids detection in the Saghro massif, Eastern Anti-Atlas, Morocco. False color composites (FCC), band ratios and principal component analysis (PCA) were employed to VNIR/SWIR and TIR data for detecting major lithological contacts and mineralized faults. The supervised maximum-likelihood (MLL) classifications and spectral angle mapper (SAM) were carried out on VNIR + SWIR data for discriminating granitoid rocks (Massironi et al., 2008).

ASTER thermal infrared bands were used for identifying quartz and carbonate minerals in northern Nevada, USA (Rockwell and Hofstra 2008). Quartz Index (QI - Ninomiya et al., 2005 ) and Carbonate Index (CI - Ninomiya et al., 2005) were implemented on ASTER data for mapping hydrothermal quartz and carbonate rocks at regional and local scales, which can be host rock a wide range of metallic ore deposit types. The potential of Linear Spectral Umixing (LSU) method over ASTER VNIR and SWIR data has been investigated for targeting and quantification of mineral content in limestone and bauxite rich areas in Southern India (Sanjeevi 2008). The results, not only targeted limestone and bauxite accurately, but also estimated the quality of these deposits.

New ASTER band ratios, including (2 + 4)/3, (5 + 7)/6 and (7 + 9)/8 have been produced for mapping ophiolitic rocks (serpentinites, metagabbros and metabasalts) in the Central Eastern Desert of Egypt (Amer et al., 2010). Principal Component Analysis (PCA) was also applied for discriminating between ophiolitic rocks and grey granite and pink granite. The achieved results from field works verified the accuracy and potential of these methods using ASTER data for lithological mapping in arid and semi-arid regions. ASTER VNIR and SWIR band combinations have been analyzed using decorrelation stretch algorithm for identifying areas containing hydrothermally altered rocks and tufa deposition at Pyramid Lake, Nevada, USA (Kratt et al., 2010).

ASTER VNIR/SWIR and TIR bands have been used for mapping albite granite in the Central Eastern Desert of Egypt. Running band ratio, band combinations and Quartz Index (QI) allowed the discrimination of albite granite from the other rock types in the study area (Aboelkhair et al., 2010). Rotation Variant Template Matching (RTM - van der Werff et al., 2007) algorithm has been employed to ASTER data to detect pre-defined lithological boundaries in the Fars group formation and the NW-SE trending Garangan anticline, southwest of Iran. Their results indicated that the main output of the RTM algorithm allowed the detection of areas having target minerals consisting of gypsum-calcite and calcite-illite in different rock units (evaporites, marly limestone and standstone) in the study areas (Salati et al., 2011).

Gypsum outcrops have been mapped using ASTER imagery in Tuzgolu basin, south Ankara, Turkey (Oztan and Suzen 2011). They used band ratio, decorrelation stretch, feature-oriented principal component analysis and thermal indices for mapping evaporate minerals. The methods used were successful in mapping evaporates. They defined sulfate index (SI) using thermal bands of ASTER that yielded a more refined result than the other methods used. The results of the methods have been proven in the field and through laboratory analysis (X-ray diffraction (XRD) and Analytical Spectral Devices (ASD)).

ASTER data have been used to map the Buraburi granite in the Himalaya of Western Nepal (Bertoldi et al., 2011). They applied a GIS-based visual analysis of RGB false color composite, band ratio and Relative absorption Band Depth (RBD), as well as principal component analysis (PCA) on the six SWIR and the five TIR masked bands. The methods discriminated among Fe-Oxide, Fe-Mg-OH, Al-OH and CO_3_ using VNIR/SWIR bands, and between silicates and carbonates using TIR bands.

ASTER data have been utilized for lithological mapping in the Oscar Coast area, Graham Land, Antarctic Peninsula (Haselwimmer et al., 2011). Matched Filter (MF - Harsanyi et al. 1994) method was applied to ASTER VNIR/SWIR and TIR data to discriminate the major lithologic groups within the study area as well as delineation of hydrothermal alteration zones. The results have shown the discrimination of most of the major lithologic units, and the delineation of propylitic and argillic alteration zones associated with volcanic rocks. The outcomes have enabled important revisions to the existing geological map of the study area.

Mixture Tuned Matched Filtering (MTMF - Boardman et al., 1995; Boardman, 1998) algorithm have been applied to VNIR and SWIR bands of ASTER for identifying mineral components of soils covering western region of the state of Sao Paulo, Brazil (Vicente and Filho 2011). The method identified Kaolinite, montmorillonite, gibbsite and hematite in the tropical soils. The results validated using reflectance spectroscopy and X-ray diffractometry (XRD).

Spectral Feature Fitting (SFF) algorithm has been implemented to VNIR + SWIR and TIR ASTER data to map lithological units in the Neyriz ophiolite, southwestern Iran (Tangestani et al. 2011). They applied the algorithm using laboratory reflectance and emittance spectra of rock samples as end-members for discriminating ophiolite rock units. Altered dunite and pridotites, pillow lava, gabbro, marble and rediolarite cherts were discriminated successfully using the algorithm applied to the ASTER SWIR data, which were typically better than those achieved using ASTER VNIR + SWIR and TIR data. Results compared well with the geological map of the study area and field observations.

ASTER and HyMap data have been used for mineral and lithological mapping in the Kap Simpson complex, central East Greenland. Matched Filtering (MF) algorithm was applied to map jarosite, ferric oxides and Al-OH clays minerals using ASTER VNIR/SWIR data. Lithological units have been identified by applying color composite of the ASTER TIR bands. The intergration of the results with HyMap data produced useful information for mineral exploration activities in the Arctic regions of central East Greenland (Bedini 2011).

ASTER data have been utilized for lithologic mapping of the Khanneshin carbonatite volcano, southwest of Kandahar, Afghanistan (Mars and Rowan 2011). They used false color composite image, band ratio, the logical operator algorithms (Mars and Rowan 2006) and matched filter methods to VNIR-SWIR and TIR bands of ASTER. Quaternary carbonate rocks within the volcano were identified and discriminated from Neogene ferruginous polymitic and argillite rocks. Their results showed the distribution of calcitic and ankeritic carbonatites, agglomerates, contact metamorphosed rocks, argillic and sandstone and iron-rich sandstone using VNIR-SWIR bands. Widespread silica and carbonate rocks, mafic-rich rock and sediment were identified using TIR bands. Results provided image-based map of rocks and minerals that are consistent with available geologic map of the study area.

Support Vectro Machine (SVM - Vapnik 1995) algorithm has been used as an automated lithological classification method to ASTER data for geological mapping study in Proterozoic Aravalli-Delhi Orogen located in the state of Rajasthan, northwestern India (Yu et al., 2012). Principal component analysis and independent component analysis were used as image enhancement techniques for lithological discrimination. Several datasets extracted from ASTER data products, including ASTER level 3 and digital elevation model, as well as areomagenetic data were used as input datasets for SVM algorithm. The method performed well in discriminating rocks types, in particular, granite, quartizite and mica schist, although it was useful in classification of vegetation, water bodies and dry steams. The results compared well with maximum likelihood classifier (MLC) method, but SVM algorithm provide higher accuracy in classification of independent validation samples as well as similarity with the available bed-rock lithological map.

During recent years, ASTER data have been used widely for mapping regional hydrothermal alteration zones associated with porphyry copper and epithermal gold mineralization (Pour and Hashim 2012a). The capability of the ASTER data for mapping the hydrothermally altered rocks and the unaltered country rocks associated with porphyry copper mineralization has been evaluated in the Cuprite mining district in Nevada, USA (Rowan et al., 2003). Matched Filtering (MF) technique was used for identifying the surface distribution of hydrothermal alteration minerals. The results indicated that spectral reflectance differences in the nine bands of visible near infrared through the shortwave infrared (0.52 to 2.43 μm) can provide subtle spectral information for discriminating main hydrothermal alteration mineral zones. A silicified zone, an opalized zone, an argillized zone and the distribution of unaltered country rock units have been identified.

Principal Component Analysis (PCA) has been applied on ASTER VNIR and SWIR bands in order to target key alteration minerals associated with epithermal gold deposits in Los Menucos, Patagonia, Argentina (Crosta et al., 2003). PCA was applied to selected subsets of four ASTER bands according to the position of characteristic spectral absorption features of key hydrothermal alteration mineral end-members such as alunite, illite, smectite and kaolinite in the VNIR and SWIR regions. Their results revealed that PCA technique can extract detailed mineralogical spectral information from ASTER data by producing abundance images of selected minerals. The distribution of hydrothermally altered rocks consisting of phyllic, argillic and propylitic alteration zones and hydrothermally silicified rocks associated with Cu-Au mineralization has been mapped using spectral analysis of VNIR + SWIR and TIR ASTER bands in the Reko Diq, Pakistan (Rowan et al., 2006). Numerous high-potential areas of porphyry copper and epithermal or polymetallic vein-type mineralization were identified based on argillic and phyllic alteration patterns in the Zargros magmatic arc, Iran (Mars and Rowan 2006). They used the logical operator algorithms on ASTER defined band ratios to illustrate distinctive patterns of argillic and phyllic alteration zones associated with Eocene to Miocene intrusive igneous rocks, as well as known and undiscovered porphyry copper deposits.

Mixture Tuned Matched Filtering (MTMF) method has been applied to ASTER SWIR data to provide regional and local information on the spatial distribution of hydrothermal alteration zones associated with epithermal gold mineralization at the Somún Curá Massif, Patagonia, Argentina (Ducart et al. 2006). Matched Filtering (MF) method has been employed to EO-1 Hyperion and ASTER data to extract abundance images for gold-associated lithological mapping in southeastern Chocolate Mountain, California, USA (Zhang and Pazner 2007). The assessment of matched filtering score index indicated the ASTER data has good capability in discrimination and classification of rock types. Although, the Hyperion data can produce better accuracy than ASTER data, the lithologic information extracted from ASTER image data is mostly similar with Hyperion results. The better availability and vast spatial coverage of ASTER data make it more suitable for regional scale lithological mapping.

Band ratios, certain color band combinations and the Spectral Angle Mapper (SAM) method have been used for mapping hydrothermal alteration minerals associated with Infiernillo porphyry copper deposit using ASTER data covering the San Rafale Massif, southern Mendoza Province, Argentina (Di Tommaso and Rubinstein 2007). The hydrothermal alteration anomalies for predicting Cu-Au mineral resources have been delineated using ASTER data covering Oyu Tolgoi, Mongolia (Yujun et al., 2007). Gold-related lithologic and alteration minerals have been detected using ASTER data in the south Chocolate Mountains area, California, USA (Zhang et al., 2007).

Several ASTER false color composites have been used to visualize lithological units and structural lineaments associated with stratiform Cu mineralization at Lufukwe, Lufilian Foreland, Democratic Republic of Congo (El Desouky et al. 2008). ASTER data have been used for alteration zone enhancement related to porphyry copper mineralization in northern Shahr-e-Babak, Iran (Tangestani et al., 2008). Mineral alteration zones associated with gold deposits in the Takab area, north-west Iran have been mapped using ASTER data (Moore et al., 2008). High potential gold mineralization areas have been detected using ASTER data covering Abu-Marawat, North-Eastern Desert of Egypt (Gabr et al. 2010).

Hydrothermal alteration minerals have been identified using SWIR bands of ASTER for porphyry copper and epithermal gold exploration in east Zanjan, northern Iran (Azizi et al., 2010). Spectral Feature Fitting (SFF), Spectral Angle Mapper (SAM) and Binary Encoding (BE) were applied to recognize hydrothermal alteration mineral classes such as chlorite-carbonate, calcite-dolomite-magnesite, kaolinite-smectite and alunite-illite. Two main alteration zones, including propylitic and phyllic-argillic were discriminated using identified alteration mineral classes.

New prospects of porphyry copper deposits have been detected using VNIR/SWIR ASTER data in the NW-SE trending Central Iranian Volcanic Belt, southeastern Iran (Pour and Hashim 2011a). The performance of principal component analysis, band ratio and minimum noise fraction transformation has been evaluated for the Visible and Near Infrared (VNIR) and the Shortwave Infrared (SWIR) subsystems of ASTER data. The image processing methods indicated the distribution of iron oxides and vegetation in the VNIR subsystem. Hydrothermal alteration mineral zones associated with porphyry copper mineralization identified and discriminated based on distinctive shortwave infrared radiation properties of the ASTER data in a regional scale. These methods identified new prospects of porphyry copper mineralization in the study areas. The spatial distribution of hydrothermal alteration zones has been verified by in-situ inspection, X-ray diffraction (XRD) analysis and spectral reflectance measurements.

Linear Spectral Unmixing (LSU) and and Mixture Tuned Matched Filtering (MTMF) algorithms implemented on VNIR/SWIR bands of ASTER for mapping alteration minerals related to copper mineralization in the Sarduiyeh area, southeastern Kerman, Iran (Hosseinjani and Tengestani 2011). They identified three groups of alteration minerals consisting of pyrophylite-alunite, sericite-kaolinite and chlorite-calcite-epidote. Their results showed high overall accuracy, and have been confirmed by field observation and X-ray diffraction (XRD) analysis of field samples.

ASTER, ETM^+^ and airborne magnetic-radiometric data have been used for hydrothermal alteration mapping at Sar Cheshmeh porphyry copper deposit, southeastern Iran. Principal Component Analysis (PCA), band ratio and the Spectral Angle Mapper (SAM) methods were used to map hydrothermally altered rocks. Result showed that ASTER SWIR-derived images enhanced hydrothermally altered rocks using PCA (PCs 2 and 3) and band ratios (4/9 and 7/6) methods. SAM classification image detected sericite, chlorite and calcite with a total accuracy of 71.3%. ETM^+^ data were used to enhance iron oxides rich areas using the PC5 image. Potassic alteration recognized well using airborne magnetic-radiometric data (Rajendran et al. 2011).

ASTER and Phased Array L-band Synthetic Aperture Radar (PALSAR) data have been used for mapping lithology and gold-related alteration zones in the Um Rus area, Central Eastern Desert of Egypt (Amer et al., 2012). Principal component analysis and band ratioing were applied on VNIR + SWIR bands of ASTER to discriminate lithological units. Spectral Angle Mapper (SAM) and Spectral Information Divergence (SID) classification methods were used to detect alteration minerals consisting of sericite, calcite and clay minerals associated with mineralized granodiorite. Their field verification work indicated that the image processing methods were capable in lithological and alteration mineral mapping.

The application of spectral image processing methods to ASTER data for mapping hydrothermal alteration zones associated with porphyry copper mineralization and related host rock has been investigated in the southeastern segment of the Urumieh-Dokhtar volcanic belt of Iran (Pour and Hashim 2012b). Spectral transform approaches, namely principal component analysis, band ratio and minimum noise fraction were used for mapping hydrothermally altered rocks and lithological units at regional scale. Spectral mapping methods, including spectral angle mapper, linear spectral unmixing, matched filtering and mixture tuned matched filtering were applied to differentiate hydrothermal alteration zones associated with porphyry copper mineralization such as phyllic, argillic and propylitic mineral assemblages. Spectral transform methods enhanced hydrothermally altered rocks associated with the known porphyry copper deposits and new identified prospects using shortwave infrared (SWIR) bands of ASTER. These methods showed the discrimination of quartz rich igneous rocks from the magmatic background and the boundary between igneous and sedimentary rocks using the thermal infrared (TIR) bands of ASTER at regional scale. Spectral mapping methods distinguished the sericitically- and argillically-altered rocks (the phyllic and argillic alteration zones) that surrounded by discontinuous to extensive zones of propylitized rocks (the propylitic alteration zone) using SWIR bands of ASTER at both regional and district scales. Results have proven to be effective, and in accordance with the results of field surveying, spectral reflectance measurements and X-ray diffraction (XRD) analysis.

Band ratioing, principal component analysis (PCA), false-color composition (FCC), and frequency filtering (FFT-RWT) have been applied to ASTER and ETM^+^ data to improve the visual interpretation for detailed mapping of the Gebel Egat area in South Estern Desert of Egypt (Zoheir and Emam 2012). By compiling field, petrographic and spectral data, controls on gold mineralization have been assessed in terms of association of gold lodes with particular lithological units and structures.

ASTER data have been also successfully used for massive sulfide, magnetite and chromite exploration. Propylitic alteration zone and gossan associated with massive sulfide mineralization have been distinguished by using ASTER (4/2, 4/5, 5/6) band ratio images covering the Neoproterozoic Wadi Bidah shear zone, southwestern Saudi Arabia (Velosky et al., 2003). ASTER data have been used for exploring areas of hydrothermal alteration and gossan related to massive sulfide deposits in the Nuqrah area, Saudi Arabia (Assiri and Mousa, 2008). Simple color composite was developed using bands 4, 6 and 9 of ASTER to detect iron-rich cap or gossan and hydrothermal alteration zones. Band ratios of 5/7, 5/4, and band 2/1 in RGB were also used to map hydrothermal alteration zones and gossan in the Nuqrah area.

ASTER data have been utilized for distinguishing sodic-calcic, potassic and silicic-phyllic alteration patterns associated with hydrothermal iron oxide deposits in the Chadormalu paleocrater, Bafq region, Central Iran (Moghtaderi et al. 2007). Iron ores deposits and associated lithology have been discriminated using new ASTER band ratios and principal component analysis in high grade granulite region of Salem, Southern Peninsular India (Rajendran et al. 2011). ASTER band ratios (1 + 3)/2, (3 + 5)/4 and (5 + 7)/6) in a RGB color composite were generated for mapping iron ore deposits. Principal component analysis was used to discriminate the iron ores and garnetiferrous pyroxene granulite rock. Results showed that the image processing methods can produce useful information for discriminating the different rock types and iron ores (magnetite quartzite deposits) using ASTER data.

Chromite bearing mineralized zones have been detected using VNIR and SWIR bands of ASTER in Semail Ophiolite Massifs of the northern Oman Mountains. Serpentinized harzburgite rocks containing chromites have identified by applying decorrelated stretching, different band ratioing and principal component analysis (Rajendran et al., 2012).

### Mapping lithology and ore minerals exploration using EO1 data

Some studies were carried out using EO1 data (ALI and Hyperion) for lithological mapping and ore mineral exploration. The capacity of VNIR and SWIR subsystems of Hyperion data for mineral mapping has been evaluated at Mountain Fitton, South Australia (Cudahy et al., 2001). The Hyperion derived mineral map indicated spatially coherent mineral distributions consistent with the geology map as well as superimposed alteration. The results showed the capability of Hyperion data and the spectral power for mineral mapping especially in SWIR bands. Mixture Tuned Matched Filtering (MTMF) method implemented to Hyperion data, including all available bands with particular attention to SWIR region (2000–2400 nm) for hydrothermal alteration mineral mapping at Panorama, Western Australia (Cudahy and Barry 2002). Two types of white mica (Al-rich and Al poor), chlorite and pyrophyllite have been recognized. The resultant Hyperion derived mineral maps of white mica abundance and Al-chemistry were correlated well with the corresponding HyMap white mica maps and the published geologic maps.

The performance of Airborne Visible/Infrared Imaging Spectrometer (AVIRIS) data has been compared with Hyperion data for mineral mapping in Cuprite, Nevada and northern Death Valley, south-central California/Nevada, USA (Kruse et al., 2003). Visual comparison of the Hyperion and AVIRIS mineral maps for both case studies indicated that Hyperion generally identified similar minerals and produced similar mineral mapping results to AVIRIS. However, the lower signal-to-noise of the Hyperion data in SWIR region has affected the ability to extract characteristic spectra and identify individual minerals. Results established that the Hyperion SWIR (2.0-2.4 μm) data can be used to produce useful mineralogical information (Kruse et al., 2003). ALI, Hyperion and ASTER data have been used for alteration mineral mapping in the Central Andes between Volcan Socompa and Salar de Liullaillaco located in the border region between Chile and Argentina (Hubbard et al., 2003).

Hubbard and Crowley (2005) utilized ALI, ASTER and Hyperion data for mineral mapping in a volcanic terrane area of the Chilen-Bolivian Altiplano. ASTER and ALI channels were co-registered and jointed to produce a 13-channel reflectance cube spanning the visible to shortwave infrared radiation (0.4-2.4 μm). Minimum Noise Fraction (MNF) transformation, Pixel Purity Index (PPI) and *n-*Dimensional Visualizer were applied to identify spectral end-members. Spectral Angle Mapper (SAM) and Linear Spectral Unmixing (LSU) were applied to map altered rocks using extracted spectral end-members. Results showed that the Hyperion data was only marginally better for mineral mapping than the merged ALI + ASTER datasets.

Hyperion and AVIRIS data have been used for district-level mineral surveying associated with epithermal gold mineralization in the Los Menucos District, Rio Negro, Argentina (Kruse et al., 2006). VNIR and SWIR bands of Hyperion and AVIRIS were analyzed to identify iron oxides, clay minerals and carbonates. Hematite, goethite, dickite, alunite, pyrophyllite, muscovite/sericite, montmorillonite, calcite and zeolites were identified in the study area using Hyperion and AVIRIS data. Field reconnaissance verification and spectral measurements showed the accuracy of hyperspectral mapping results.

Hydrothermally altered rocks and a Percambrian metamorphic sequence have been identified using Hyperion data at and around the Alid volcanic dome, at the northern Danakil Depression, Eritrea (Gersman et al., 2008). They discriminated the different types of rock groups by using unsupervised and supervised classification approaches. The ability of the Hyperion to detect ammonium spectral signature was reported. The existence of ammonium in hydrothermally altered rocks within the Alid dome has been confirmed by previous studies.

Hyperion and ASTER data have been used for mineral mapping in the Pulang, Yunnan Province, China (Bishop et al., 2011). ASTER data have been utilized to locate target areas characterized by hydrothermal alteration minerals and Hyperion data for detailed mineral mapping. Principal component analysis and band ratioing methods were applied to ASTER data to detect target areas characterized by argillic alteration, iron oxides and sulfate minerals. Spectral Angle Mapper (SAM) and Mixture Tuned Matched Filtering (MTMF) were implemented on Hyperion data to discriminate mineral species in the target areas. Iron oxide minerals consisting of hematite, goethite, limonite and jarosite were detected using VNIR bands of Hyperion. Sericite, kaolinite, montmorillonite, muscovite and illite were discriminated using SWIR bands of Hyperion. Results indicated that the combination of multispectral and hyperspectral data can be advantageous for mineral exploration in remote areas with limited or unavailable primary information (Bishop et al., 2011).

Earth Observing-1 (EO-1) ALI and Hyperion data have been used to extract the geological and mineralogical information for identifying hydrothermal alteration zones associated with porphyry copper deposits in southeastern segment of the Central Iranian volcanic belt, SE Iran (Pour and Hashim 2011d). A band ratio derived from image spectra (4/2, 8/9 and 3 in RGB) has been developed to identify lithological units and hydrothermally altered rocks using ALI data in a regional scale. AIG-Developed Hyperspectral Analysis processing methods were tested on the shortwave infrared bands of Hyperion for mapping mineral assemblages in hydrothermal alteration zones associated with porphyry copper ore deposits. The methods produced image map of spectrally predominant minerals in alteration zones using Hyperion data. Therefore, phyllic, argillic and propylitic alteration zones were significantly discriminated from surrounding country rock. The spatial distribution of identified hydrothermal alteration zones has been confirmed by spectral reflectance measurements, XRD analysis and in-situ inspection. Their results indicated that lithological units, hydrothermally altered rocks and hydrothermal alteration zones associated with porphyry copper mineralization can be accurately mapped by ALI and Hyperion data at both regional and district scales (Pour and Hashim 2011d).

## Conclusions

This paper reviews applications of the ASTER, ALI and Hyperion data as a tool for mapping lithology and ore minerals exploration. The comparison between results revealed that: (i) ASTER SWIR bands allow key distinctions to be mapped between various clay, chlorite, epidote and sulfate mineral types, and TIR bands have sufficient capability for detecting quartz and carbonate minerals; (ii) ALI has sufficient spectral resolution in the VNIR wavelength range to discriminate several important ferric-iron oxide minerals, and SWIR bands are useful for regional alteration mineral mapping; (iii) Hyperion is useful for calibrating ASTER and ALI data, and can also be used for evaluating the mineral mapping results and producing spectrally predominant minerals map. Application of the ASTER, ALI, and Hyperion data in the field of lithological mapping and ore minerals exploration are summarized in Table 4.

**Table 4:** **Application of the ASTER, ALI, and Hyperion data in the field of lithological mapping and ore minerals exploration**

Geological applications	Lithological and structural mapping	Porphyry copper and epithermal gold	Chromite and magnetite	Mineral components of soils	Massive sulfide
ASTER	Identification the variety of igneous felsic, mafic-ultramafic rocks, metamorphic rocks, sedimentary rocks, and ophiolite components. Faults, fractures, anticlinal or synclinal faults, and lithological boundaries.	Identification and discrimination of gossan, argillic, advanced argillic, phyllic, potassic, propylitic and silicic zones.	Detection of serpentinized harzburgite.Identification of magnetite quartzite, sodid-calcic zone, potassic and silicic-phyllic patterns.	Identification the variety of iron oxide/hydroxid-es minerals, clay minerals chlorite and epidote mineral groups, carbonate minerals, and silicate minerals.	Detection of gossan, propylitic, and silicic zones.
ALI	Detection the variety of igneous, metamorphic, sedimentary, and ophiolite rock complex. Faults, fractures and lithological boundaries at regional scale.	Identification of hydrothermally altered rocks at regional scale.	Identification of serpentinized harzburgite. Iron oxide/hydrox-ides minerals.	Detection and discrimination the variety of iron oxide/hydroxid-es minerals. Identification of clay minerals at regional scale.	Detection of gossan.
Hyperion	Chemical composition of different types of rocks and mineral abundance in the rocks at district scale. Detection of lithological boundaries, faults, fractures, and joints at district scale.	Detection the abundance of specific minerals in hydrothermal alteration zones at local and district scales.	Detection of serpentinized harzburgite. Identification of magnetite quartzite, sodid-calcic zone, potassic and silicic-phyllic patterns at district scale.	Detection the abundance of iron oxide/hydroxid-es minerals, clay minerals chlorite and epidote mineral groups, carbonate minerals in the soil at local and district scales.	Detection of gossan and propylitic zones at local and district scales.

The overlap coverage of the EO1 (ALI and Hyperion) and EOS/Terra (ASTER) data allows obtaining comprehensive information for the reconnaissance stages of ore minerals exploration in virgin areas and future lithology mapping. The integration of the ASTER, ALI, and Hyperion data has great ability to identify hydrothermal alteration zones and lithological mapping at both regional and district scales. The applied algorithms that used to map, enhance and discriminate lithology and hydrothermal alteration mapping were reviewed in detail by Pour and Hashim (2012a). All of the techniques and achievements that reviewed in this paper emphasize on the efficacy of ASTER, ALI, and Hyperion data for future purposes in the field of ore minerals exploration and lithological mapping.

In conclusion, the integration of ALI, ASTER and Hyperion imagery can be an effective technique for mapping a variety of minerals characteristic of hydrothermally altered rocks for exploring ore deposits in remote areas of the earth, where existing geologic and other ground truth information is restricted.
